# Femoral neck fracture combined with anterior dislocation of the femoral head: injury mechanism and proposed novel classification

**DOI:** 10.1186/s12891-021-04703-w

**Published:** 2021-09-21

**Authors:** Jichao Liu, Zhengwei Li, Jie Ding, Bingzhe Huang, Chengdong Piao

**Affiliations:** 1 Department of Orthopedics, The Second Hospital of Jilin University, 218 Ziqiang Road, Nanguan Street, Changchun, 130041 Jilin Province China; 2 Department of Stomatology, The Affiliated Hospital of Changchun University of Traditional Chinese Medicine, Changchun, Jilin China

**Keywords:** Femoral neck fracture, Hip dislocation, Classification

## Abstract

**Background:**

Femoral neck fracture combined with anterior dislocation of the femoral head is very rare. To our knowledge, there is no classification system yet for this rare form of injury, and the injury mechanism of femoral neck fracture combined with obturator head dislocation has not been described in the literature. In this study, we systematically reviewed the literature and the cases treated in our hospital, and identified and classified all injury types according to the injury mechanism of femoral neck fracture combined with anterior dislocation of the femoral head. Further, based on the experience of treating a patient with femoral neck fracture and obturator dislocation of the femoral head, a theoretical hypothesis was proposed for the injury mechanism of this rare type of injury.

**Methods:**

A comprehensive search was conducted on PubMed, WOS, CNKI database. These fractures were classified according to the dislocation site and injury mechanism (one injury or two injuries).

**Results:**

1891 articles were initially identified through PubMed and other databases, and after bibliographic research, study screening, and removing duplicates, 1455 articles were selected. After applying the exclusion criteria, a total of 18 full-text articles describing femoral neck fractures combined with anterior dislocation of the femoral head. Different dislocation sites have different injury mechanisms. Our classification system, to the best of the authors’ knowledge, allowed us to include all types of femoral neck fractures combined with anterior dislocation of the femoral head from the literature. According to the proposed classification system, the morphological features of femoral neck fracture combined with anterior dislocation of the femoral head can be accurately conveyed between doctors.

**Conclusions:**

All injury patterns can likely be identified using the proposed classification system. This can help avoid confusion in the nomenclature of femoral neck fractures combined with anterior dislocation of the femoral head and help surgeons to more accurately detect lesions, thereby guiding surgical treatment.

## Introduction

Femoral neck fracture is a commonly encountered fracture in the orthopedic clinic and accounts for about 3–5% of all fractures [1, 2]. However, femoral neck fracture combined with anterior dislocation of the femoral head is a very rare type of injury [3, 4]. This type of injury is often the result of high-energy vertical shear violence in the hips or lower limbs [5]. These high-energy injuries in young patients may result in loss of reduction, fracture nonunion, and avascular necrosis of the femoral head [6–8]. Epstein et al. [9, 10] defined that the most important factors causing traumatic anterior dislocation of the hip are forced flexion, abduction, and external rotation; in this position, the femoral neck and the greater trochanter violently hit the acetabular edge. As a result, the femoral head is pried out of the acetabulum and pushed forcefully toward the front of the acetabulum. During this process, if the hip joint is in flexion, it will cause obturator hip dislocation, and if the hip joint is in extension, it will result in pubic hip dislocation [11]. For more complex injuries such as femoral neck fractures combined with obturator dislocations of the femoral head, the mechanism of injury is still very poorly understood. We believe that femoral neck fractures combined with obturator dislocations of the femoral head are the result of two injuries.

The Pauwels classification was first described in 1935 and is still frequently used to classify femoral neck fractures. There are three classification cut-off values: <30 degrees, 30–50 degrees, and >50 degrees [12]. Using these classifications, the shear force over the fracture site can be determined. The theoretical principle behind this classification is that fractures with a more vertical fracture line experience greater shearing forces and thus present a greater risk for fracture healing complications [13]. Collinge et al. [14] calculated the fracture morphology of 136 high-shear angle femoral neck fractures in young adults aged < 50 years old and found the following: 1. The average Pauwels angle of the fracture was 60 degrees, and the average external rotation deformity was 44 degrees; 2. Femoral neck comminution was identified in 96% cases, mostly centered in the inferior (94%) and posterior (82%) quadrants; 3. The average shortening was 1.8 cm; 4.63% patients presented with the beak-shaped bone process at the proximal end of the fracture. However, we found that most patients with femoral neck fracture combined with anterior dislocation of the femoral head did not show these characteristics. Moreover, in current clinical practice, the most commonly used classification for femoral neck fractures combined with femoral head dislocation has a simple description, namely “anterior dislocation of the hip with femoral neck fracture” or “obturator hip dislocation with fracture of the femoral neck” Thus far, there is no exclusive fracture classification for femoral neck fractures combined with anterior dislocation of the femoral head, and there is no common mode of communication and evaluation. At present, the Brumbback classification can be used to describe the fracture classification of femoral neck fracture combined with anterior dislocation of the femoral head (Table 1) [15]. The description provided by the above classification system fails to describe the injury patterns localized to the femoral neck fracture combined with anterior dislocation of the femoral head. Because no classification system can accurately judge the detailed position of the anterior dislocation of the femoral head and the degree of displacement, typically no recommendations for fracture treatment are provided, and these are pure descriptions.

**Table 1:** Brumback Classification system for hip dislocation and femoral head fracture

Type I	FRACTURE OF INFEROMEDIAL ASPECT OF FEMORAL HEAD
A	Minimal or no acetabular fracture, stable hip joint
B	Relevant acetabular fracture, unstable hip joint
Type II	FRACTURE OF SUPEROMEDIAL ASPECT OF FEMORAL HEAD
A	Minimal or no acetabular fracture, stable hip joint
B	Relevant acetabular fracture, unstable hip joint
Type III	HIP DISLOCATION WITH FEMORAL NECK FRACTURE
A	No femoral head fracture associated
B	Femoral head fracture associated
Type IV	ANTERIOR HIP DISLOCATION WITH FEMORAL HEAD FRACTURE
A	Indentation type
B	Transchondral shear type
Type V	CENTRAL HIP DISLOCATION WITH FEMORAL HEAD FRACTURE

In this study, we attempted to classify femoral neck fractures with anterior dislocation on the basis of Pauwels fracture classification, combined with the injury mechanism of hip fracture with dislocation. Different fracture types are described according to the injury mechanism and dislocation site to improve clinicians’ understanding of the typical injury patterns of this site. Because the injury mechanism of posterior dislocation is completely different from anterior dislocation, it is not included in our classification system. Based on our experience in the diagnosis and treatment of femoral neck fracture with femoral head dislocation, we have proposed a theoretical hypothesis on the injury mechanism of femoral neck fracture with obturator dislocation.

## Materials & methods

Because these fractures are very rare, it is difficult to study a large number of patients. Therefore, in this study, on the basis of a systematic review of the literature on femoral neck fracture with anterior dislocation of the femoral head, and combined with the related cases treated in our hospital, we developed a new classification of femoral neck fracture with dislocation of the femoral head. Additionally, we proposed a theoretical hypothesis on the injury mechanism of femoral neck fracture with obturator dislocation.

### Systematic literature search

The systematic search was conducted following the PRISMA (Preferred Reporting Items for Systematic Reviews and Meta-Analyses) guidelines in September 2021 using PubMed databases, Web of Science databases and CNKI databases. A general search was conducted using the following terms: (((((((((((Dislocation, Joint) OR (Dislocations, Joint)) OR (Joint Dislocation)) OR (Luxation Erecta)) OR (Inferior Dislocation)) OR (Inferior Dislocations)) OR (Joint Subluxations)) OR (Joint Subluxation)) OR (Subluxation, Joint)) OR (Subluxations, Joint)) AND (((Femoral Neck Fracture) OR (Femur Neck Fractures)) OR (Femur Neck Fracture))). Articles written in languages other than English and Chinese were excluded. References from the articles were reviewed to confirm completeness of the identified literature.

### Exclusion and inclusion criteria

**Fig. 1:**
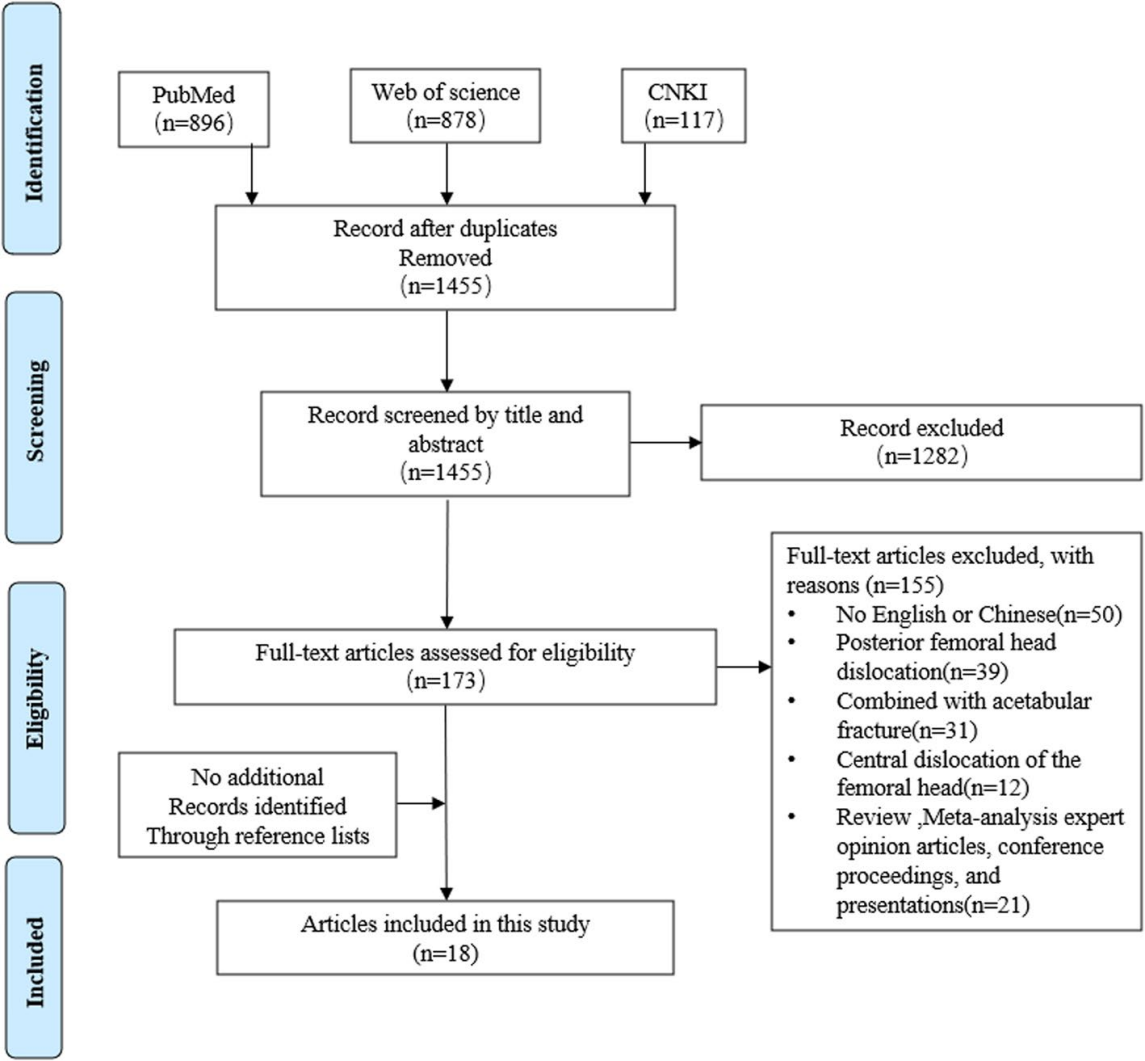
PRISMA flowchart of literature selection

The inclusion criteria of the literature require that, whether case report or clinical study, the patients described in the literature must have a femoral neck fracture combined with an anterior dislocation of the femoral head. Due to different injury mechanisms, fractures with acetabular fracture or central dislocation of the femoral head are not included in our inclusion criteria. Exclusion criteria for literature were as follows: articles not written in English or Chinese; review, Meta-analysis, and expert opinion articles, conference proceedings, and presentations; femoral neck fracture combine with an acetabular fracture or central dislocation or posterior of the femoral head. Use the PRISMA guidelines to search independently (Fig. 1). One author (LJC) conducted a literature search, and two authors (LZW, DJ) independently reviewed the search results. The titles and abstracts of all search results were reviewed. Obtain full-text articles to identify studies that meet the inclusion and exclusion criteria. If there is a disagreement, please consult the senior author (PCD).

### Data collection

A standardized data table was developed for collecting data from various literature.

Literature data collection included (1) patient demographic data, such as age, gender;(2) injury mechanism;(3) dislocation type;(4) we also measured the Puawels angle for the literature with imaging data;(5) treatment;(6) surgical approach;(7) the prognosis of the patient.

### Data analysis

Based on the injury history and radiographic data of each patient, we analyzed the injury mechanism of femoral neck fracture combined with anterior dislocation of the femoral head; We then counted the type of dislocation of the femoral head in each patient and then classified it according to the mechanism of injury and the direction of dislocation; Finally, we analyzed the effect of different treatments and surgical approaches on the outcomes of femoral neck fractures associated with dislocation.

## Results

### Literature search

Electronic database search found 1891 articles. After eliminating duplicate publications, 1455 articles were further screened. Then we excluded articles that did not have an English or Chinese version(n=52). After the title and abstract were screened for relevance, 1282 articles were considered irrelevant according to the inclusion and exclusion criteria. After applying the inclusion and exclusion criteria, 121 articles were included. Of the 103 publications, 39 were posterior femoral head dislocation; 31 were combined with acetabular fracture; 12 were central dislocation of the femoral head, and 21 were reviews or curative analysis. Ultimately, 18 articles including 18 patients fulfilled the inclusion criteria and were included in this study (Table 2).

**Table 2:** Review of patients with femoral neck fracture combined with anterior dislocation of the femoral head

Study	Year	Sex/Age (years)	Injury cause	Dislocation type	Puawels angle	Treatment	Approach	Outcome
Ayman [3]	2017	M/28	Traffic accident	Obturator subluxation	76°	Cannulated screws	Watson Jones	Full recovery at 2 years
Jain.S et al. [16]	2015	M/17	Traffic accident	Obturator subluxation	51°	DHS	Modified Hardinge	Full recovery at 6 months
Dummer et al. [17]	1999	M/48	Traffic accident	Obturator subluxation	54°	THA	N/A	Full recovery at 8 months
Esenkaya et al. [18]	2002	F/39	Traffic accident	Anterior pubic dislocation	52°	THA	Lateral approach	Internal rotation disorder at 5 years,no avascular necrosis
Izquierdo et al. [19]	1994	F/17	Traffic accident	Obturator	60°	Cannulated screws	N/A	Full recovery at 6 months
Fan et al. [20]	2003	M/41	Smashing injury	Obturator subluxation	N/A	Cannulated screws + Bone flap transplantation	Modified Smith-Petersne	Full recovery at 9 months
Li et al. [21]	2011	M/40	Smashing injury	Anterior pubic dislocation	N/A	THA	N/A	Lost to follow-up
Song et al. [22]	2003	M/51	Fall while running	Pelvic	56°	Artificial Femoral Head Replacement	N/A	Lost to follow-up
Zhou et al. [23]	2012	M/41	Smashing injury	Subpubic dislocation	62°	Cannulated screws	Moore approach	Full recovery at 8 months
Kang et al. [24]	1985	M/50	Traffic accident	Subpubic dislocation	50°	Artificial Femoral Head Replacement	N/A	Slight discomfort at 3 years, no avascular necrosis
Liu et al. [25]	1993	M/40	Traffic accident	Anterior pubic dislocation	N/A	Scale stitch + Bone flap transplantation	Smith-Petersne	Full recovery at 9 months
Sadler et al. [11]	1985	M/27	Traffic accident	Anterior pubic dislocation	77°	DHS	Watson-Jones	Osteonecrosis of the femoral head at 6 weeks,full recovery after Judet-Meyers muscle-pedicle grafting procedure
Jain et al. [26]	2017	M/32	Traffic accident	Obturator subluxation	63°	THA	Watson-Jones	Full recovery at 6 weeks
Allagui et al. [27]	2013	M/40	Traffic accident	Anterior pubic dislocation	60°	DHS	Watson-Jones	Full recovery at 12 weeks
McClelland et al. [28]	1986	M/28	Traffic accident	Obturator subluxation	64°	THA	Posterolateral incision	Full recovery at 6 months
Hu et al. [29]	2018	F/34	Traffic accident	Obturator	85°	Cannulated screws combined with medial buttress plate	Modified Smith-Petersne	Full recovery at 9 months
Pankaj et al. [30]	2011	M/33	Traffic accident	Obturator	52°	THA	Posterolateral incision	Full recovery at 18 months
Fina et al. [31]	1970	M/10	Traffic accident	Anterior pubic dislocation	N/A	THA	N/A	Full recovery at 11 months

*DHS* Dynamic hip screw, *THA* Total Hip Arthroplasty

### Fracture patterns

**Fig. 2:**
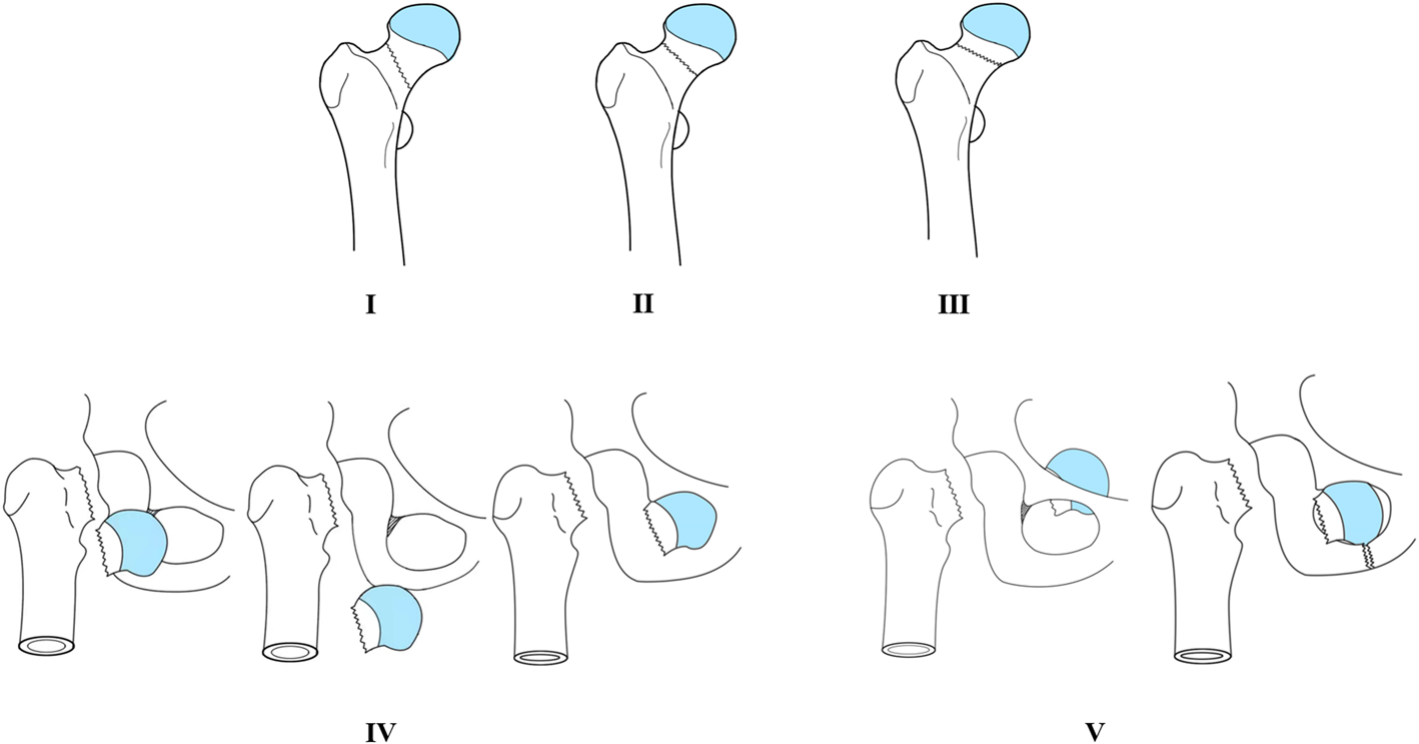
Comprehensive injury-mechanism-based classification system. (I)Type I: Pauwels angle is <30°, and shows no dislocation of the femoral head. (II)Type II: Pauwels angle is between 30°–50° and shows no dislocation of the femoral head. (III)Type III: Pauwels angle is >50° and shows no dislocation of the femoral head. (IV)Type IV: Pauwels angle is >50°, and is accompanied by anterior dislocation (obturator subluxation, anterior pubic dislocation, and subpubic dislocation); this is the result of only one impact. (V)Type V: Pauwels angle is >50° and accompanied by obturator dislocation of the femoral head or pelvic dislocation. This is the result of two injuries

The distribution of injury was as follows: obturator subluxation (n=6, 33.3%) [3, 16, 17, 20, 26, 28]; anterior pubic dislocation (n=6, 33.3%) [11, 18, 21, 25, 27, 31]; obturator dislocation (n=3, 16.7%) [19, 29, 30]; subpubic dislocation (n=2, 11.1%) [23, 24]; and pelvic dislocation (n=1, 5.6%) [22]. The mainly cause of injury was traffic accident (n=15, 83.3%). According to the imaging data and injury process, we classified the types of femoral neck fractures and femoral head anterior dislocations (Table 3 AND Fig. 2).

**Table 3:** Classification system of Femoral neck fracture combined with anterior dislocation of femoral head

Type I	Pauwels angle <30°, and no dislocation of the femoral head
Type II	Pauwels angle is between 30°-50°, and no dislocation of the femoral head
Type III	Pauwels angle >50°, and no dislocation of the femoral head
TypeIV	Pauwels angle >50°, accompanied by anterior dislocation
a	anterior pubic dislocation
b	obturator subluxation
c	subpubic dislocation
TypeV	Pauwels angle>50°, accompanied by anterior dislocation
a	obturator dislocation of the femoral head
b	pelvic dislocation

### Surgical treatments

Artificial hip replacement was applied in 9 patients, 2 of whom were underwent artificial femoral head replacement, and the remaining 7 patients underwent THA. Five patients received cannulated screws as the initial treatment, including one with bone flap transplantation and one with medial buttress plate to counteract the vertical shear force. DHS was utilized in 3 patients. In addition, Scale stitch combined with bone flap transplantation was applied in the remaining 1 patient.

### Prognosis

In addition to the absence of prognostic results in two patients, we recorded the prognosis of others. Most of the patients undergoing artificial arthroplasty were reported to have a good prognosis. Unfortunately, one patient developed internal rotation disorder in the fifth year after operation, and another patient developed hip discomfort in the third year after operation, but showed no signs of avascular necrosis of the femoral head. Patients who received cannulated lag screws were reported to have a good prognosis. One patient developed osteonecrosis of the femoral head in the sixth week after DHS. Interestingly, he fully recovered after the Judet-Meyers muscle-pedicle grafting procedure.

## Discussion

Femoral neck fractures combined with anterior dislocation of the femoral head are very rare [17, 18, 32]. It is usually caused by a high-energy injury [33], most often seen in traffic accidents among young adults. Dislocation of the hip is considered an orthopedic emergency and should be restored as soon as possible within 6–8 h of the trauma [34]. At present, there is no detailed classification for this kind of injury. While there are existing hip dislocation classifications such as the Brumback classification [15], the description is not limited to anterior dislocation, and hence no existing classification system can accurately judge the detailed location of anterior dislocation of the femoral head and the degree of displacement. Therefore, it is not convenient for doctors to accurately convey the characteristics of these fractures [35]. To address this issue, we searched the cases of femoral neck fracture combined with anterior femoral head dislocation in our hospital and other aforementioned databases. By analyzing the imaging data and injury mechanism of these patients, we proposed a novel classification system based on the injury mechanism for femoral neck fracture combined with anterior femoral head dislocation. We believe that this new classification provides a common mode of communication and evaluation. Moreover, to our knowledge, the injury mechanism of femoral neck fractures combined with obturator dislocation have not been clarified so far in the literature. By treatment of a case of femoral neck fracture combined with obturator dislocation of the femoral head, we have put forth a theoretical hypothesis for the injury mechanism of this type of injury.

Through our literature search, we finally identified and included 18 patients in this study. The main cause of injury was due to traffic accidents. We performed Pauwels angle measurement and statistics on patient’s radiographies (14 cases) with imaging data according to the modified Pauwels angle measurement method of Wang et al. [36]. Taking the central axis of the femoral shaft as the reference datum line, make a vertical line through the upper edge of the femoral head to intersect it, and then make the fracture line intersect the vertical line of the upper edge of the femoral head, then the angle between the fracture line and the vertical line of the upper edge is the Pauwels angle. Pauwels angle measurement results showed that Pauwels classification of femoral neck fractures of these patients was type III and the average angle was 60 degrees. It can be seen that in this injury mode, femoral neck fractures are caused by huge vertical shear stresses through the femoral neck.

The Pauwels angles of Type I and Type II are P<30° and between 30°–50°, and they are not accompanied by femoral head dislocation. For young adults or active elderly people, these two types of fractures usually require the use of multiple cancellous lag screws or dynamic hip screws (DHS) for in situ fixation [37, 38]. Compared with DHS, multiple lag screws offer the advantage of a minimally invasive technique, shorter operation time, and sufficient fixation to achieve the most stable fracture pattern [39].

Type III: Pauwels angle is >50° and shows no dislocation of the femoral head. This type of fracture is caused by high-energy vertical shear stress. Owing to the huge vertical shear force, the incidence of adverse postoperative complications is high [40, 41].

Type IV: Pauwels angle is >50°, and is accompanied by anterior dislocation (obturator subluxation, anterior pubic dislocation, and subpubic dislocation); it is the result of only one impact. The injury mechanism of this type of fracture is that the hip joint is in the position of external pronation and extension and is placed on the front seat back. When the knee is suddenly braked, the upper part of the femoral head is stressed by the knee, and the force continues to be transmitted along the femoral shaft, causing the upper and outer part of the femoral head to hit the acetabulum. At this time the femoral neck undergoes considerable shear stress, resulting in a vertical shear fracture of the femoral neck. If the force does not dissipate, the distal end of the fractured end continues to move toward the acetabulum, resulting in the femoral head being pried out of the acetabulum. The femoral head is dislocated to the front and eventually blocked by the sartorius muscle.

**Fig. 3:**
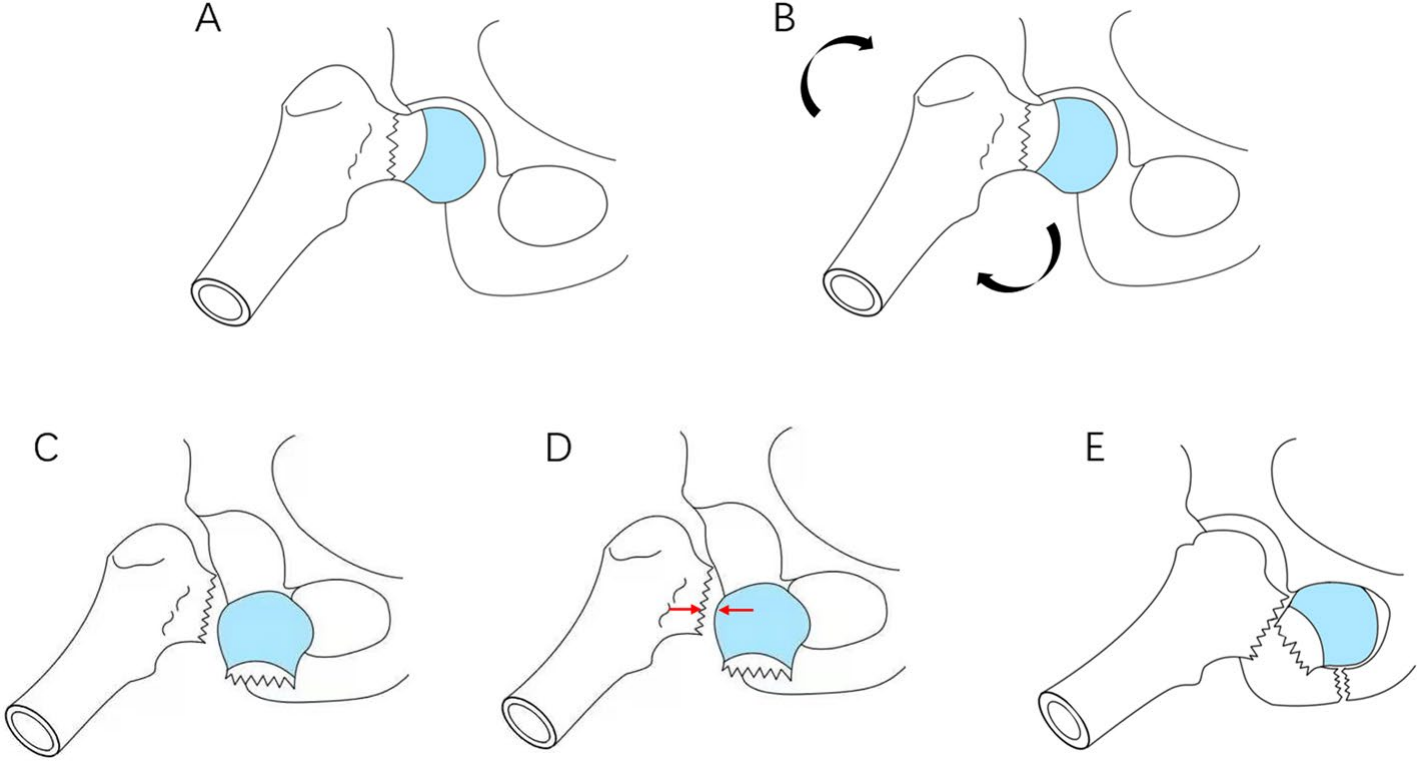
Injury mechanism of femoral neck fracture combined with obturator dislocation. The inner side of the proximal femur hits the outer and upper parts of the femoral head. After the second impact, the femoral head dislocated to the obturator through the sartorius tunnel and was blocked by the pubic branch, resulting in a fracture of the inferior pubic branch, which eventually caused the femoral head to be embedded in the obturator

Type V: Pauwels angle is >50° and accompanied by obturator dislocation of the femoral head or pelvic dislocation [22, 42]. This is the result of two impact. This type of injury is not categorized as Type IV, because the femoral head is dislocated into the obturator or pelvis. Type V is the result of two injuries to the hip, which is different from the first injury in Type IV. Based on the Type IV injury mechanism, after the femoral head is dislocated to the front and blocked by the sartorius muscle, the hip is re-hit by the side of the hip. This violence is conducted through the medial side of the distal femoral stump. In the dislocated femoral head, the head hits the inferior pubic branch. After the inferior pubic branch is fractured, eventually dislocated and stuck in the obturator foramen (Fig. 3). Based on the experience of treating a patient with femoral neck fracture combined with obturator dislocation of the femoral head, the basis of this theoretical hypothesis is as follows: 1. The inner side of the proximal femur hits the outer and upper parts of the femoral head. During the operation, a 1 cm× 1 cm defect on the outer and upper parts of the femoral head can be found. This is caused by the impact of the proximal femur during the second impact. 2. The “sartorius tunnel” can be detected during the operation. This tunnel is the muscle space between the four muscles. The inner and outer sides of the tunnel are composed of different muscles. On the outside, the anterior wall is the sartorius muscle and the posterior wall is the medial femoris muscle. The outer edges of these two muscles form the entrance of the sartorius tunnel. On the inside, the anterior wall is the iliopsoas muscle and the back wall is the pubis muscle. The inner edges of these two muscles form the exit of the tunnel. After the second impact, the femoral head dislocated to the obturator through the sartorius tunnel and was blocked by the pubic branch, resulting in a fracture of the inferior pubic branch, which eventually caused the femoral head to be embedded in the obturator. 3. Fracture of the inferior pubic branch, which is caused by the impact of the femoral head. If there is no second impact, the fracture is not strong enough to cause a fracture of the inferior pubic branch. 4. The direction of the vertical shear stress of the femoral neck is inconsistent with the direction of dislocation of the femoral head into the obturator, which must be the result of two injuries. Medda et al. [43–45] reported several cases of anterior hip dislocation with pubic fracture, but no femoral neck fracture. We believe that this injury was caused by only one violence. After the femoral neck fracture, the second violence caused the proximal femur to hit the femoral head, which in turn caused the femoral head to hit the pubic bone; this eventually resulted in femoral neck and pubic branch fractures and femoral head dislocation. This injury process is similar to the process of playing golf; hence, we refer to this combination of fracture and dislocation as a “Golf fracture” (Fig. 3E).

Types III, IV, and V fracture ends are displaced significantly, and the blood supply of the femoral head is severely damaged. In the elderly, total hip replacement is often performed to obtain postoperative stability and early recovery of mobility [46]. However, in relatively young people (<60 years old), regardless of whether the fracture type is non-displaced or displaced, head-saving surgery is essential [47]. These kinds of injuries usually occur in young patients. The average age of the patients is 35.7 (17–51) years. At present, there is no consensus on whether to choose open reduction and internal fixation or THA for this special injury [48]. All cases included in this study underwent open reduction and various types of internal fixation or THA. This result indicates the fact that once the femoral neck fracture is combined with dislocation, closed reduction is not a viable option [27]. Closed reduction of femoral fracture combined with dislocation will destroy the remaining blood vessels, thereby affecting revascularization and leading to poor prognosis [49]. The treatment options for patients included in this study were THA (n=7), femoral head replacement (n=2), inverted triangular cannulated screws (n=5), DHS (n=3), and cannulated screws combined with medial support steel plate (n=1) [19]. According to reports by Esenkaya et al., after they used THA to treat this special injury, the patient developed internal rotation disorder [18]. Kang et al. performed femoral head replacement for their patient with this special injury, but the patient developed discomfort in the affected hip after the operation [21, 24]. Sadler et al. reported an anterior dislocation of the hip joint with ipsilateral base neck fracture that was fixed with hip screw and plate reduction. Unfortunately, their patient developed avascular necrosis and required pedicle bone grafting [11]. Ayman Mohammad El Masry applied cannulated screws to fix the fracture, after which the patient developed pain on movement [3]. Among the remaining patients, four were lost to follow-up, and the prognosis of the remaining patients was good. This shows that the prognosis of this type of injury is not positive. In young adults (<60 years old), regardless of whether the fracture type is non-displaced or displaced and considering the younger age and expected higher functional requirements, arthroplasty procedures are not ideal given the young age and expected higher functional demands [50]. Femoral head preservation should be the first choice. Owing to the huge shear stress, the treatment of Pauwels type III fractures in young patients is difficult and the prognosis is poor [14]. At present, the methods of internal fixation of femoral neck fractures include cannulated screws, DHS (with or without additional anti-rotation screws), and cephalomedullary nails [51–53]. However, the surgical fixation of these fractures has a high failure rate, ranging from 20% to 80% [54–56]. In order to improve the stability of fixation, based on previous research, Mir et al. [57] in 2015 proposed the concept of medial buttress plate for the treatment of femoral neck fractures in young patients. The medial buttress plate can clamp the fracture fragments, counteract the shear force, and convert it into compression force, which is transmitted to the cannulated screws or DHS [58]. Kunapuli et al. [59] first studied the strength of augmented versus nonaugmented with medial buttress plate for stabilizing vertical shear femoral neck fractures. Their results showed that the medial buttress plate could increase the maximum load by 83%. Jia Li et al. [60] used finite element analyses (FEA) to compare the outcomes of using a combination of medial buttress plate with cannulated screws vs. using cannulated screws alone. The combined use of medial buttress plates could provide better stability, which indicates better healing of femoral neck fractures. According to reports by Ye et al., the use of cannulated screws combined with medial buttress plate for the treatment of femoral vertical neck fractures can effectively reduce fracture nonunion and surgical failure rates, and improve postoperative joint function [61–63]. Therefore, cannulated screws combined with medial buttress plate has become the most popular treatment for young adult patients with Pauwels III fractures.

If the condition of the patient permits, the fracture of the femoral neck with anterior dislocation of the femoral head must be reduced with the least delay possible [64]. There is a high risk of nonunion and avascular necrosis of the femoral head after femoral neck fracture [65]. Among the patients included in this study, only one case of femoral head necrosis occurred. One of the reasons for such a low incidence is that most patients (n=13) received surgical treatment within 10 h of the injury. If the reduction is within 6 hours, the risk of avascular necrosis will be reduced from 40% to 10% [66, 67]. Sendtner and Manninger et al pointed out that if the reduction exceeds 6 hours, hemorrhage in the joint capsule will cause an increase in pressure, which is not conducive to the healing of the fracture [68–70].

The choice of surgical approach is also critical. In this review, four surgeons [3, 11, 17, 26] chose the W-J approach; three surgeons [20, 71] chose the S-P approach or its modified approach; and two surgeons each chose the lateral approach [16, 18] and the posterolateral approach [23, 28]. The remaining literature did not describe the surgical approach in detail. The anterior approach of the hip joint is also called the S-P approach. This approach passes through the nerve interface between the sartorius muscle and the tensor fascia lata muscle through the outer layer of the surrounding muscles, which can safely expose the hip joint. The modified S-P approach utilizes the caudal extent of the standard S-P interval distal to the anterosuperior iliac spine and parallel to the palpable interval between the tensor fascia lata and the sartorius muscle [72]. The anterolateral approach of the hip (W-J approach) is a commonly used approach for THA. This approach passes through the plane between the tensor fascia lata muscle and the gluteus medius, which can clearly show the acetabulum and is safe for reaming of the femoral shaft [73]. Surgeons usually choose the W-J or modified S-P approach, and this decision usually depends on comfort and familiarity. The modified S-P approach utilizes the intermuscular and internervous plane, wherein the surgeon can directly access the femoral neck for visualization and address other pathological problems, such as femoral head fractures [74]. Ye et al. compared these two surgical approaches and found that the modified S-P approach is superior to the W-J approach as in the former, the surgeon can directly inspect and palpate the femoral neck and surrounding structures [61]. Therefore, for such abnormal injuries, surgeons should consider the modified S-P approach.

## Conclusions

In this study, we carried out a retrospective analysis of cases of femoral neck fracture combined with anterior dislocation of the femoral head in order to classify this type of fracture pattern. We found that types V had the worst prognosis, followed by types IV. We believe that based on this classification system, doctors can easily distinguish this type of fracture pattern from other types and avoid the naming confusion associated with femoral neck fractures and anterior dislocations; furthermore, it could help surgeons to accurately communicate the characteristics of fractures with colleagues, thereby guiding surgical treatment.

## Data Availability

All data generated or analyzed during this study are included in this article.

## Abbreviations

THA: Total Hip Arthroplasty
DHS: Dynamic Hip Screw
S-P: Smith–Petersen
W-J: Watson–Jones
